# Supplementary material for: The ubiquitin-like modifier FAT10 interferes with SUMO activation
Source: Nat Commun. 2019 Oct 1;10:4452. doi: 10.1038/s41467-019-12430-z (PMC6773726; doi:10.1038/s41467-019-12430-z)
Supplement: Supplementary file 1 — Supplementary Information [file 41467_2019_12430_MOESM1_ESM.pdf]

The ubiquitin-like modifier FAT10 interferes with SUMO activation

Aichem et al.

Supplementary Information

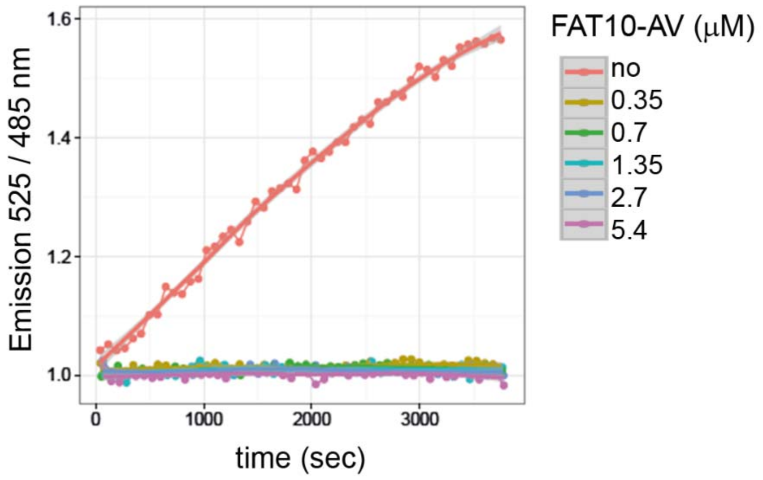

Supplementary Fig. 1 FAT10-AV inhibits RanGAP1-tail SUMOylation. A FRET-based *in vitro* SUMOylation assay with the SUMO substrate RanGAP1-tail (RanGAP), fused to CFP, and SUMO-1, fused to YFP, as described in Fig. 3a. The indicated amounts of FAT10-AV were added to the reaction containing CFP-RanGAP, AOS1/UBA2, UBC9 and YFP-SUMO-1, and the FRET signal was measured over a time course of 1 hour.

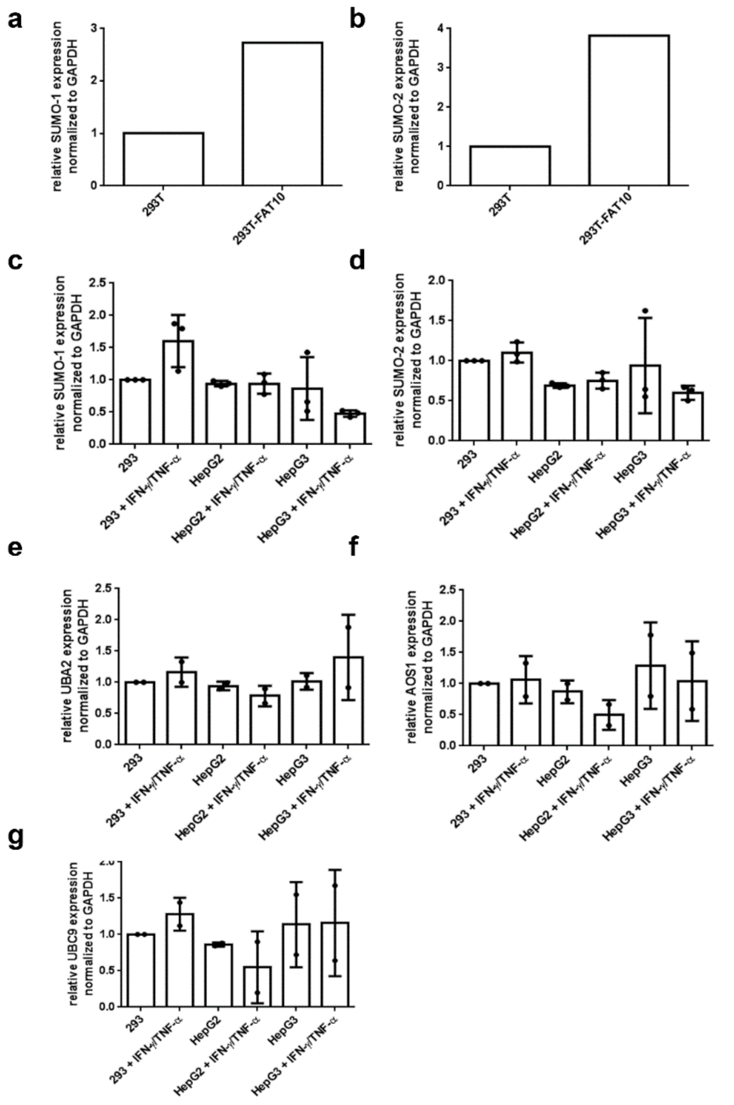

Supplementary Fig. 2 Expression of FAT10 or treatment with IFN-γ/TNF-α does not change the mRNA levels of *SUMO-1*, *SUMO-2*, *AOS1/UBA2* or *UBC9*. (a)+(b): *SUMO-1* and *SUMO-2* mRNA expression level in HEK293T wildtype or stable FLAG-FAT10 expressing HEK293T cells (293T-FAT10). (c)-(g): mRNA expression levels of indicated genes for HEK293, HepG2 and HepG3 cells, treated or not for 24 hours with IFN-γ/TNF-α. Data represent the mean of n= 3 ((c) and (d)) or n=2 ((e), (f) and (g)) independent experiments. All mRNA levels were normalized to the levels of the housekeeping gene glyceraldehyde 3-phosphate dehydrogenase (*GAPDH*). The value of untreated HEK293 cells was set to unity and all other values were calculated accordingly. Shown is the mean +/- s.e.m..

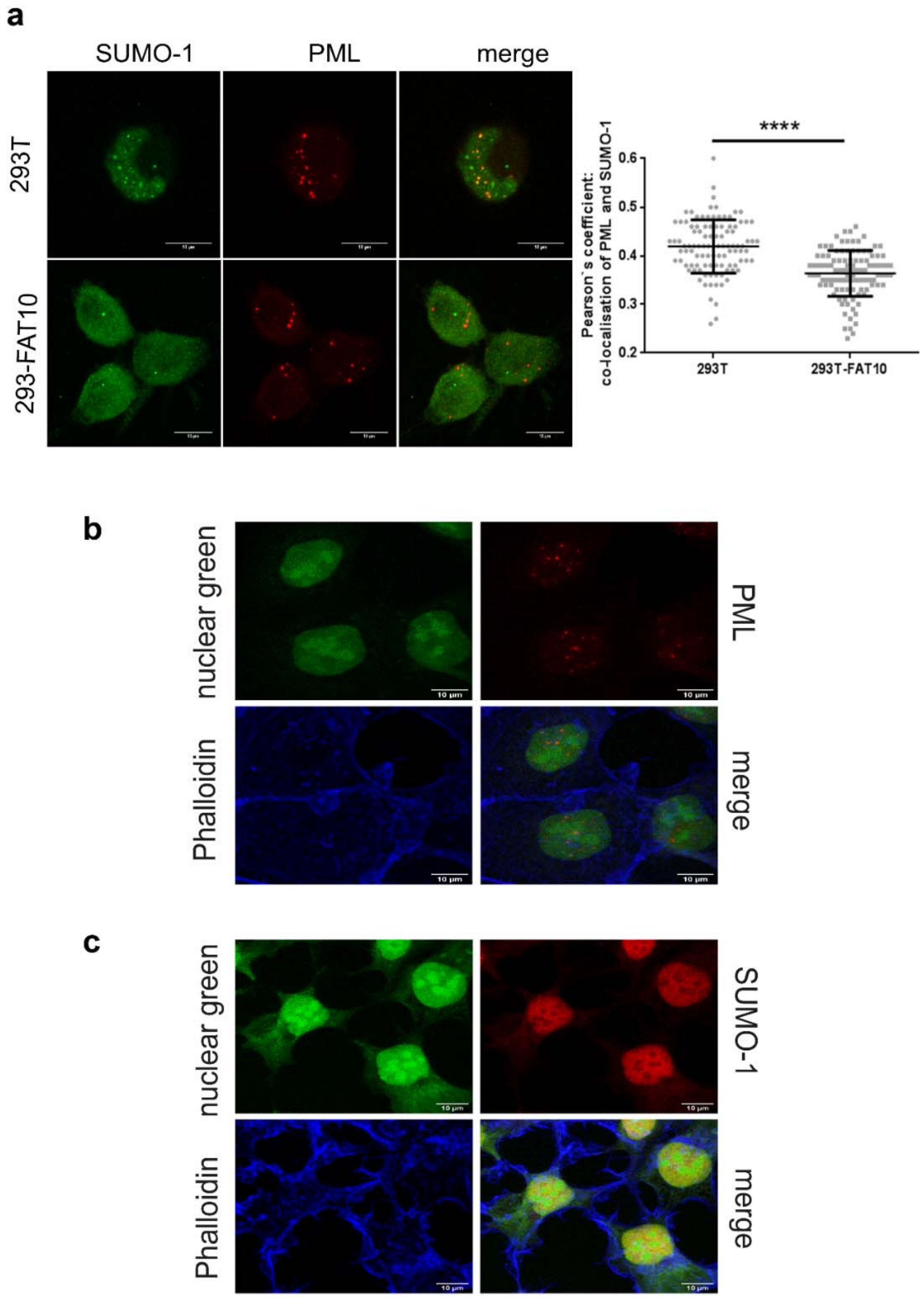

Supplementary Fig. 3 Less SUMO-1 and PML co-localization in presence of FAT10. **a** (left panel): Confocal microscopy pictures of HEK293T wildtype (293T) and of stably FLAG-FAT10 expressing HEK293T cells (293-FAT10). Cells were stained with antibodies reactive against SUMO-1 or PML, as indicated. The scale bar represents 10 μm. (Right panel): Quantification of the Pearson`s coefficient,

representing the probability of co-localization of SUMO-1 and PML. n=105 cells of each cell type out of two independent experiments were used for calculations. Significance was calculated using an unpaired, non-parametric Mann-Whitney test. A two-tailed P-value of < 0.0001 was considered to be highly statistically significant (****). **b** and **c** Both, PML and SUMO-1 localize to the nucleus. Confocal microscopy pictures of HEK293 wildtype cells, stained with Nuclear Green DCS1, Phalloidin and SUMO-1 or PML-reactive antibodies.

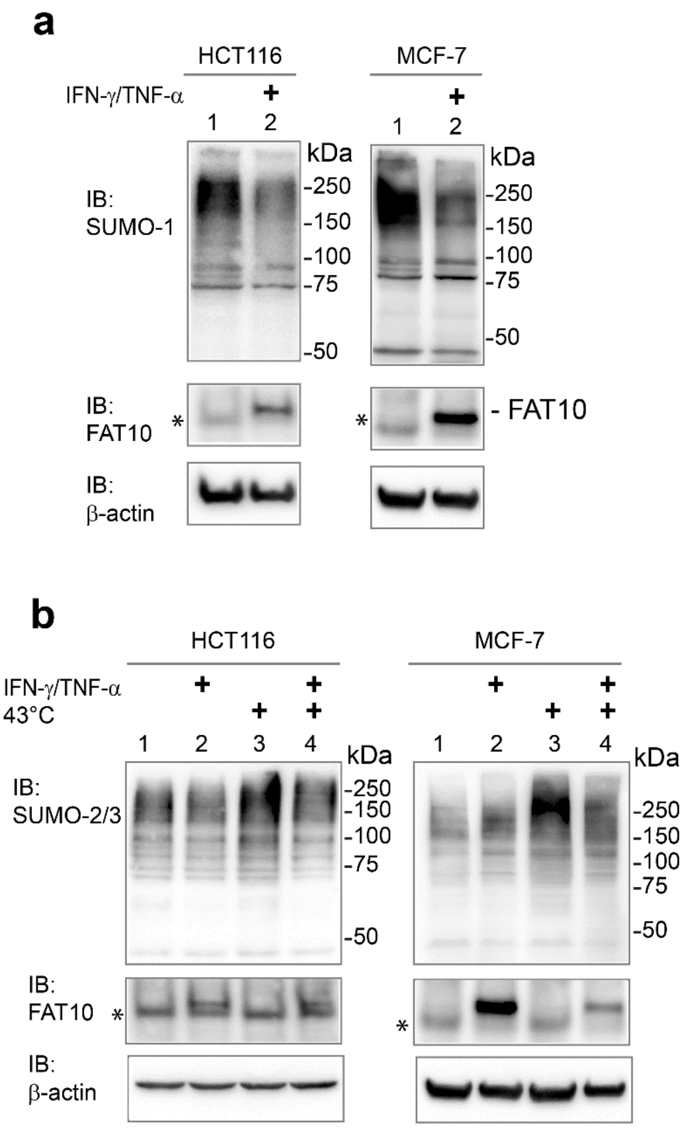

Supplementary Fig. 4 Less SUMO-1/2/3 conjugates in presence of endogenous FAT10. **a, b** Western blot analyses of SUMO-1 or SUMO-2/3 conjugates in crude cells lysates of HCT116 or MCF-7 cells, prepared in presence of 10 mM NEM. Endogenous FAT10 expression was induced with IFN-γ/TNF-α for 24 hours, as indicated. **b** Where indicated, cells were additionally treated with a 43°C heat shock before harvesting to induce SUMO-2/3 conjugation. Proteins were separated on 10% polyacrylamide gels (SUMO-1/2/3 staining) or 4-12% Bis/Tris gradient gels (Invitrogen) (FAT10 and β-actin staining) and subjected to western blot analysis using the antibodies indicated. β-actin was used as loading control. Asterisks mark unspecific background signals. Shown is one representative out of two (HCT116) or three (MCF-7) independent experiments with similar outcomes.

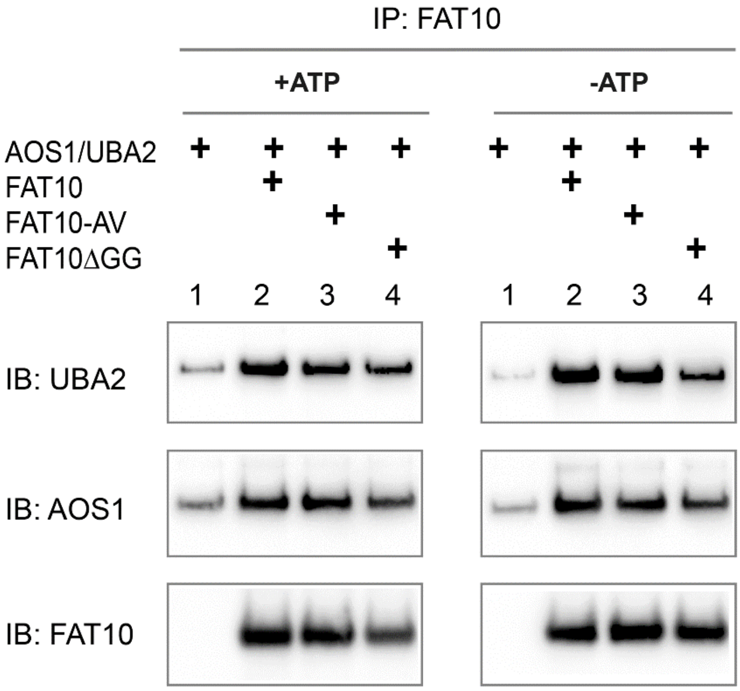

Supplementary Fig. 5 All FAT10 variants interact ATP-independently with AOS1/UBA2. *In vitro* co-immunoprecipitation experiment using recombinant AOS1/UBA2 and FAT10 variants as described in Fig. 5a. Immunoprecipitation was performed in presence or absence of ATP, as indicated, using a monoclonal FAT10-reactive antibody (clone 4F) and subsequent Western blot analysis with the antibodies indicated.

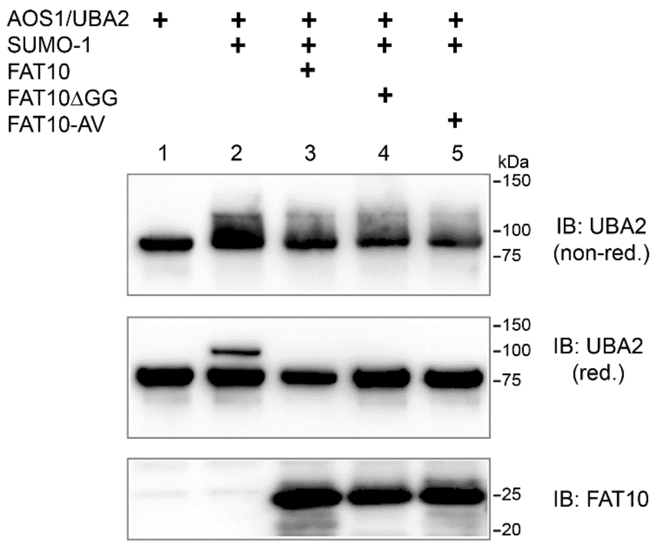

Supplementary Fig. 6 Inhibition of SUMO-1 activation in presence of FAT10 variants. Western blot showing an *in vitro* SUMO activation assay in presence of different FAT10 variants. The assay was performed for 30 minutes at 37°C and analyzed under non-reducing (non-red.) or reducing (red.) (4% 2-ME) conditions. Shown is one representative experiment out of three experiments with similar outcome. Protein amounts used were 0.34 µM AOS1/UBA2; 8.3 µM SUMO-1; 11 µM FAT10/-ΔGG/-AV, each.

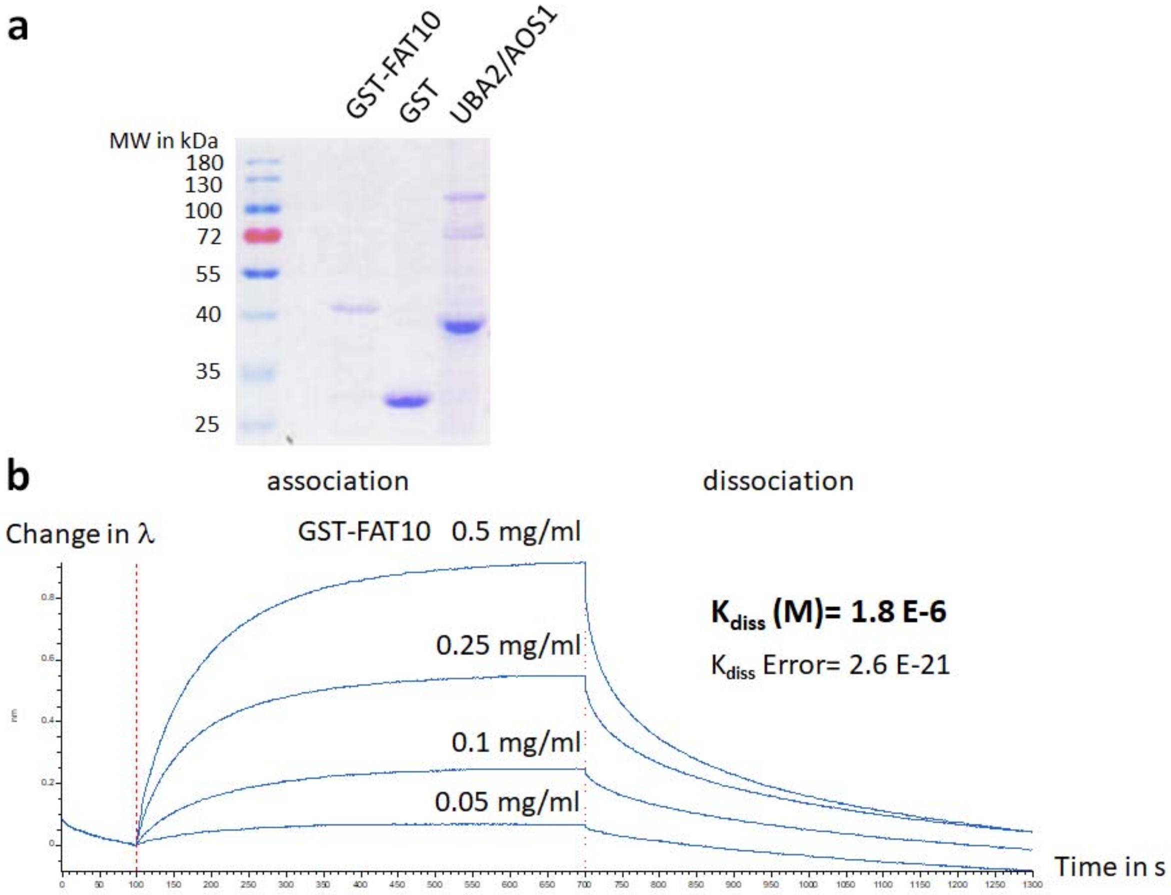

Supplementary Fig. 7 Determination of K_{diss} of GST-FAT10 binding to AOS1/UBA2. **a** Coomassie stained SDS-gel showing the different proteins (50 ng GST-FAT10, 50 ng GST, 100 ng AOS1/UBA2) used for the determination of the affinity constant K_{diss} by using the Octet system. **b** Shown are the association and dissociation curves obtained with different amounts of FAT10 (ranging from 0.5 mg/ml to 0.05 mg/ml) towards AOS1/UBA2 (always 0.1 mg/ml). The change in wavelength (nm) is plotted versus the time in seconds (s).

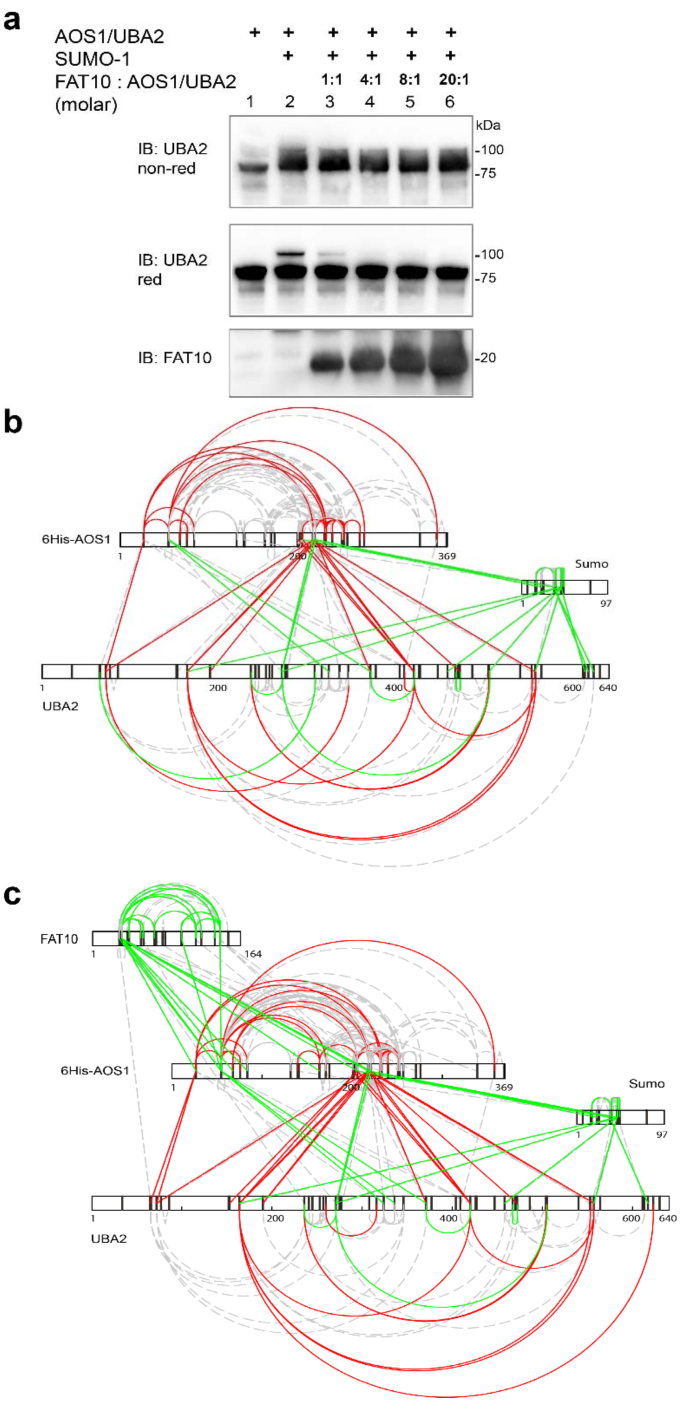
a
AOS1/UBA2
SUMO-1
FAT10 : AOS1/UBA2
(molar)
1:1 4:1 8:1 20:1
1 2 3 4 5 6
kDa
IB: UBA2
non-red
-100
-75
IB: UBA2
red
-100
-75
IB: FAT10
-20
b
6His-AOS1
1
200
369
Sumo
1
97
UBA2
1
200
400
600
640
c
FAT10
1
164
6His-AOS1
1
200
369
Sumo
1
97
UBA2
1
200
400
600
640

d

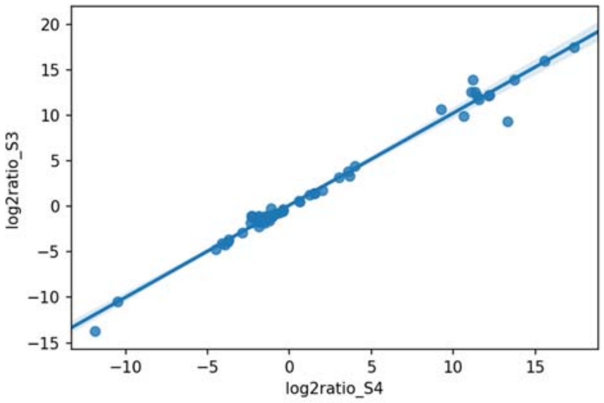

Supplementary Fig. 8 FAT10 has no discernible impact on the conformational state of AOS1/UBA2. **a** Determination of the molar excess of FAT10 versus AOS1/UBA2, needed for complete inhibition of SUMO-1 activation under *in vitro* conditions. Proteins were incubated for 10 minutes at 30°C. SUMO activation was analyzed under reducing conditions on a Western blot with a UBA2-reactive antibody. Shown is one representative experiment out of three experiments with similar outcomes. **b and c** Quantification of crosslinks in presence or absence of SUMO-1 and FAT10/SUMO-1. Q-XL-MS was used to compare crosslink abundances of AOS1/UBA2 1 (b) in presence or absence of SUMO-1 and (c) in presence or absence of SUMO-1 and FAT10. Only crosslinks that could be reproducibly quantified from the pool of identified high-confidence crosslinks in both samples (n = 3) are shown (violation = 0, p-value ≤ 0.01, lD-Score ≥ 28, see methods). Depicted in red are crosslinks that were significantly downregulated in samples with SUMO-1 or SUMO-1 and FAT10, while green links indicate significant upregulation. Crosslinks exhibiting no significant change in abundance in both samples are depicted in grey while links that could not be reliably quantified are shown with a dashed line in grey. The addition of FAT10 leads to no apparent differences in the detected crosslinks or their abundances within and between SUMO-1 and UBA2, indicating that FAT10 has also no by XL-MS discernable impact on the conformational state of the activated AOS1/UBA2. **d** Linear regression analysis of the log2 ratios of uxIDs which have been quantified in both experimental datasets (e.g. in presence or absence of SUMO-1 (S3) and in presence or absence of FAT10/SUMO-1 (S4)) results in a regression coefficient of r=0.99, indicating a strong correlation.
