# Supplementary material for: The ubiquitin-like modifier FAT10 interferes with SUMO activation
Source: Nat Commun. 2019 Oct 1;10:4452. doi: 10.1038/s41467-019-12430-z (PMC6773726; doi:10.1038/s41467-019-12430-z)
Supplement: Supplementary file 3 — Description of Additional Supplementary Files [file 41467_2019_12430_MOESM3_ESM.pdf]

Description of Additional Supplementary Files

File Name: Supplementary Data 1
Description: Intra- and inter-protein crosslinks of AOS1/UBA2 and FAT10. Data referring to Fig. 7d. Shown are the exact amino acid sequence of the crosslinked peptides and the position of the crosslinked lysine residue 'crosslinked peptide', the name of the respective protein 'protein 1' and 'protein 2', nature of the crosslink 'type', the position of the crosslinked lysine residues within the UniProt or construct sequence 'position 1' and 'position 2' (data analysis was performed on protein sequences including affinity tags. Sequence offset to UniProt sequences 6His-AOS1 + 23 aa, FAT10 -1 aa), the MS measurement in which the crosslink was identified 'raw file', 'delta S', which gives the delta score of the respective crosslink and is a measure for how close the best assigned hit was scored in regard to the second best, the 'ld-score', which is a weighted sum of different scores used to assess the quality of the composite MS2 spectrum as calculated by xQuest and the false discovery rate ('FDR') as calculated by xProphet. Crosslinks which were identified with delta-S < 0.95, lD-Score > 25 and FDR < 0.05 are listed in this table.

File Name: Supplementary Data 2
Description: Intra- and inter-protein crosslinks of AOS1/UBA2 and FAT10-AV. Data referring to Fig. 7e. Column labels are the same as described in the legend to Supplementary Data 1.

File Name: Supplementary Data 3
Description: Quantitative XL-MS for AOS1/UBA2 in presence or absence of SUMO-1. Data referring to Supplementary Fig. 8b. The table lists the relative change of different unique crosslinking sites 'uxID'. Changes in abundance are expressed as 'log2 ratio' (abundance with SUMO-1 vs. abundance without SUMO-1) and the 'p-value' indicates the regression between the two conditions. In this study, uxIDs that showed a change of log2ratio ≤ 1 and a p-value of ≤ 0.01 are considered to be significantly downregulated whereas uxIDs with a change of log2ratio ≥ 1 and a p-value of ≤ 0.01 were considered to be significantly up-regulated. The uxIDs which were consistently identified over different peak groups show 0 violations ('sum_violations'). Crosslinks that were identified only in one of the two (or multiple) samples are assigned a fractional value 'imputed values' in order to allow comparison also for crosslinks that are present only in one state: '==' indicates that a signal in both experiment and reference experiment was detected, '>=' indicates that only a signal in the experiment dataset and no signal in the reference experiment was detected and '<=' indicates that only a signal in the reference experiment could be detected. Shown are also the exact amino acid sequence of the crosslinked peptides which were taken into account for a respective uxID 'crosslinked peptide', the name of the respective protein 'protein 1' and 'protein 2', the position of the crosslinked lysine residues within the UniProt or construct sequence 'position 1' and 'position 2' (data analysis was performed on protein sequences including affinity tags. Sequence offset to UniProt sequences 6His-AOS1 + 23 aa, FAT10 -1 aa), the 'spectrum' in which the crosslink was identified as well as 'delta S', which gives the delta score of the respective crosslink and is a measure for how close the best assigned hit was scored in regard to the second best, the 'ld-score', which is a weighted sum of different scores used to assess the quality of the composite MS2 spectrum as calculated by xQuest and the false discovery rate ('FDR') as calculated by xProphet.

File Name: Supplementary Data 4
Description: Quantitative XL-MS for AOS1/UBA2 in presence or absence of FAT10 and SUMO-1. Data referring to Supplementary Fig. 8c. Column labels are the same as described in the legend to Supplementary Data 3.

File Name: Supplementary Data 5

Description: Intra- and inter-protein crosslinks of AOS1/UBA2 and FAT10 in HEPES. Column labels are the same as described in the legend to Supplementary Data 1.
