# Supplementary material for: The ubiquitin-like modifier FAT10 interferes with SUMO activation
Source: Nat Commun. 2019 Oct 1;10:4452. doi: 10.1038/s41467-019-12430-z (PMC6773726; doi:10.1038/s41467-019-12430-z)
Supplement: Supplementary file 9 — Reporting Summary [file 41467_2019_12430_MOESM9_ESM.pdf]

nature research

Corresponding author(s): Dr. Annette Aichem

Last updated by author(s): 27.08.2019

Reporting Summary

Nature Research wishes to improve the reproducibility of the work that we publish. This form provides structure for consistency and transparency in reporting. For further information on Nature Research policies, see Authors & Referees and the Editorial Policy Checklist.

Statistics

For all statistical analyses, confirm that the following items are present in the figure legend, table legend, main text, or Methods section.

n/a	Confirmed	
☐	☒	The exact sample size (*n*) for each experimental group/condition, given as a discrete number and unit of measurement
☐	☒	A statement on whether measurements were taken from distinct samples or whether the same sample was measured repeatedly
☐	☒	The statistical test(s) used AND whether they are one- or two-sided<br>*Only common tests should be described solely by name; describe more complex techniques in the Methods section.*
☒	☐	A description of all covariates tested
☒	☐	A description of any assumptions or corrections, such as tests of normality and adjustment for multiple comparisons
☐	☒	A full description of the statistical parameters including central tendency (e.g. means) or other basic estimates (e.g. regression coefficient) AND variation (e.g. standard deviation) or associated estimates of uncertainty (e.g. confidence intervals)
☒	☐	For null hypothesis testing, the test statistic (e.g. *F*, *t*, *r*) with confidence intervals, effect sizes, degrees of freedom and *P* value noted<br>*Give P values as exact values whenever suitable.*
☒	☐	For Bayesian analysis, information on the choice of priors and Markov chain Monte Carlo settings
☒	☐	For hierarchical and complex designs, identification of the appropriate level for tests and full reporting of outcomes
☐	☒	Estimates of effect sizes (e.g. Cohen's *d*, Pearson's *r*), indicating how they were calculated

Our web collection on statistics for biologists contains articles on many of the points above.

Software and code

Policy information about availability of computer code

Data collection	All MS data was collected on an Orbitrap Fusion Tribrid mass spectrometer operated with Tune (version 2.1.1465.23) (Thermo Scientific)
Data analysis	xQuest (version 2.1.3), xTract (version 1.0.2)

For manuscripts utilizing custom algorithms or software that are central to the research but not yet described in published literature, software must be made available to editors/reviewers. We strongly encourage code deposition in a community repository (e.g. GitHub). See the Nature Research guidelines for submitting code & software for further information.

Data

Policy information about availability of data

All manuscripts must include a data availability statement. This statement should provide the following information, where applicable:

- Accession codes, unique identifiers, or web links for publicly available datasets
- A list of figures that have associated raw data
- A description of any restrictions on data availability

The MS raw files, databases containing protein fasta sequences for analysis with xQuest as well as xQuest result- / xTract input- files (xtract.csv) and the xTract result files (analyzer.quant.xls) have been deposited to the ProteomeXchange Consortium via the PRIDE 56 partner repository with the dataset identifier PXD012592.

Field-specific reporting

Please select the one below that is the best fit for your research. If you are not sure, read the appropriate sections before making your selection.

☒ Life sciences ☐ Behavioural & social sciences ☐ Ecological, evolutionary & environmental sciences

Life sciences study design

All studies must disclose on these points even when the disclosure is negative.

Sample size	All experiments were performed 3-5 times, expect 2 experiments, that were repeated only twice but with exactly the same outcome. Sample sizes are described in each figure legend.<br>Crosslinked samples were prepared in biological triplicates (i.e. separately expressed and purified batches of proteins) for all investigated samples, and each of these was measured with technical duplicates.
Data exclusions	No data was excluded, only search criteria applied as described in the mansucript in order to ensure high quality MS data. (e.g. xQuest settings: identified with deltaS < 0.95 and at least one Id score ≥ 25. xTract settings: Id score ≥ 28, violations=0 and only changes that showed at least a change of log2ratio ≥ ±1 and a p-value of ≤ 0.01.A list of all identified links can be found in Supplementary Data.
Replication	In all cases, replications were successful.
Randomization	No randomization was performed as this is not common for western blot and immunoprecipitation data prevalent in this study.
Blinding	No blinding was performed as this is not common for western blot and immunoprecipitation data prevalent in this study.

Reporting for specific materials, systems and methods

We require information from authors about some types of materials, experimental systems and methods used in many studies. Here, indicate whether each material, system or method listed is relevant to your study. If you are not sure if a list item applies to your research, read the appropriate section before selecting a response.

Materials & experimental systems

n/a	Involved in the study	
☐	☒	Antibodies
☐	☒	Eukaryotic cell lines
☒	☐	Palaeontology
☒	☐	Animals and other organisms
☒	☐	Human research participants
☒	☐	Clinical data

Methods

n/a	Involved in the study	
☒	☐	ChIP-seq
☒	☐	Flow cytometry
☒	☐	MRI-based neuroimaging

Antibodies

Antibodies used	anti-FAT10 (mouse monoclonal antibody, clone 4F1, 1:50.000, Aichem et al., Nature Commun 1:13 (2010))<br>anti-FAT10 (rabbit polyclonal antibody , 1:1000, Hipp et al, Mol Cell Biol 25, 3483-3491 (2005))<br>anti-JunB (rabbit polyclonal antibody, Abcam, ab128878, 1:1000).<br>anti-β-actin (mouse monoclonal, Abcam, ab6276, 1:5000)<br>anti-FLAG-HRP (clone M2, Sigma, 1:3000, order No. A8592)<br>anti-HA-HRP (clone HA-7, Sigma, 1:4000, order No. A5795)<br>anti-SUMO-1 (rabbit polyclonal antibody, Cell Signaling #4930, 1:1000)<br>anti-SUMO-2/3 (rabbit monoclonal, clone 18H8, Cell Signaling, #4971, 1:1000)<br>anti-SAE2 (UBA2), Abcam, ab185955, rabbit monoclonal, 1:1000)<br>anti-SAE1 (AOS1) Abcam, ab185949, rabbit monoclonal, 1:5000)<br>anti-PML (Abcam ab179466, rabbit, 1:2000)
Validation	The validations of used antibodies are provided in the cited publications and on the webpages of the indicated commercial suppliers.

Eukaryotic cell lines

Policy information about cell lines

Cell line source(s)	HEK293 (ATCC® CRL-1573™)<br>HEK293T (ATCC® CRL-11268™)<br>HEK293T-FLAG-FAT10 (Spinnenhirn V, Farhan H, Basler M, Aichem A, Canaan A, Groettrup M. The ubiquitin-like modifier FAT10 decorates autophagy-targeted Salmonella and contributes to Salmonella resistance in mice. J Cell Sci 127, 4883-4893 (2014).<br>HepG2 (SIGMA/ECACC 85011430)<br>HepG3 (Guo et al., PLOS ONE 2012, Vol 7,issue 8, e43270)<br>HEK293-FAT10ko (Aichem A, Boehm AN, Catone N, Schmidtke G, Groettrup M. Analysis of modification and proteolytic

	targeting by the ubiquitin-like modifier FAT10. Methods Enzymol 619 (2019))<br>HEK293-UBA6ko (this work)<br>MCF-7 (ATCC® HTB-22™)<br>HCT116 (ATCC® CCL-247™)
Authentication	None of the cell lines used were authenticated, other than expressing the correct MHC alleles.
Mycoplasma contamination	Cells were regularly tested to be negativ for Mycoplasma infection using the MycoAlert Mycoplasma detection kit from Roche.
Commonly misidentified lines<br>(See ICLAC register)	We did not use these cell lines
